# Systematic Review of the Economics of School-Based Interventions for Dating Violence and Gender-Based Violence

**DOI:** 10.1177/10901981221138064

**Published:** 2022-12-12

**Authors:** Fraizer Kiff, Naomi Shaw, Noreen Orr, Andrew. J. Rizzo, Annah Chollet, Honor Young, Emma Rigby, Ann Hagell, Vashti Berry, Chris Bonell, G. J. Melendez-Torres, Caroline Farmer

**Affiliations:** 1 University of Exeter, Exeter, UK; 2 University of Florida, Gainesville, FL, USA; 3 University of Oxford, Oxford, UK; 4 Cardiff University, Cardiff, UK; 5 Association for Young People’s Health, London, UK; 6 London School of Hygiene and Tropical Medicine, London, UK

**Keywords:** economic evaluation, schools, dating violence, gender-based violence, resource use, systematic review

## Abstract

Dating and relationship violence (DRV) and gender-based violence (GBV) among children and young people incur a high cost to individuals and society. School-based interventions present an opportunity to prevent DRV and GBV early in individuals’ lives. However, with school resources under pressure, policymakers require guidance on the economics of implementing interventions. As part of a large systematic review funded by the National Institute for Health and Care Research (NIHR), we searched for economic evaluations and costing studies of school-based interventions for DRV and GBV. No formal economic evaluations were identified. Seven studies reporting costs, cost savings, or resource use for eight interventions were identified. The largest costs of implementing interventions were related to staff training and salaries but savings could be made by implementing interventions on a large scale. The potential cost savings of avoided DRV and GBV far outweighed the costs of implementation.

## Introduction

Dating and relationship violence (DRV) and gender-based violence (GBV) are highly prevalent among high-school-age children, experience with which has been shown to predict lifetime perpetration and victimization (Costa et al., 2015). Between a quarter and a third of adolescents experience violence within a relationship, including physical, sexual, and psychological abuse, although much higher rates have been reported in high-risk groups (Taquette & Monteiro, 2019; Young et al., 2021). GBV is also common in schools and includes sexual harassment, homophobic and transphobic bullying, and sexual assaults (Ofsted, 2021). Both victims and perpetrators experience negative effects of DRV and GBV, including mental health difficulties, low self-esteem, risky sexual behavior (Taquette & Monteiro, 2019), victims may be more likely to experience DRV and GBV as adults (Costa et al., 2015), and there may be life-changing consequences for perpetrators who enter the legal system. DRV and GBV are therefore significant public health concerns, with costly impacts for both individuals and society. Annual costs for DRV and GBV within the United Kingdom have been estimated at £66 billion and £37 billion, respectively (European Institute for Gender Equality, 2021; Oliver et al., 2019), with the majority of costs caused by the physical and mental health consequences for victims and their impact on productivity.

A large number of interventions to reduce and prevent DRV and GBV have been developed for implementation within the school setting. In the United Kingdom, state-funded schools are now required to incorporate teaching about DRV and GBV within school curricula, and educators are considering the optimum intervention content and delivery to meet the needs of students. As part of a large, nationally funded evidence synthesis project, we systematically reviewed the evidence for any intervention delivered within the school setting aimed at preventing DRV and GBV. Within this, we sought evidence for the cost-effectiveness of school-based interventions and the costs and resource use needed to implement them, as this information will be useful to policy- and decision makers. This report presents the findings of the economic outcomes reported by included studies.

## Method

An NIHR-funded systematic literature review (SLR) was conducted to assess and synthesize evidence on school-based interventions for DRV and GBV (PROSPERO: CRD42020190463). This review included a broad range of evidence including the identification of economic evaluations and studies reporting cost data (including cost savings) and resource use data.

### Search Strategy

A single search was conducted to identify all evidence types for the NIHR-funded SLR. Literature searches were conducted in July 2020 and updated in June 2021. We searched 21 databases, two trial registers, and various gray literature sources without any limit on time or study design. A range of supplementary search methods, including forward and backward citation chasing, and searches for first and last authors of included studies, were used to find additional studies. Details of literature sources and search strategies are provided in Appendix A.

### Selection Criteria

Publications reporting cost-effectiveness evaluations, cost savings, and cost and resource use data associated with interventions for DRV and/or GBV were included. Relevant study designs included both randomized controlled trials (RCTs) and observational studies. Relevant interventions were those implemented within the school setting (including out of school hours, provided these were conducted with school cohorts) and either partially or wholly aimed at changing DRV or GBV outcomes. No limit was placed on the geographical location of studies. Records identified through literature searches were screened for relevance by two reviewers at both title/abstract and full-text level.

### Outcomes

Outcomes included any cost or resource use data associated with a relevant intervention, including those required during training or implementation. Costs (savings) associated with intervention effectiveness were also included. Data were extracted in the currencies and units in which they were reported. Data extraction was conducted by one reviewer, and all extractions were reviewed by a second reviewer. Included studies were narratively synthesized.

**Impact Statement**

This systematic review has identified the most current knowledge about costs and resource use needs of school-based interventions for dating and relationship violence (DRV) and gender-based violence (GBV) and provides learnings about how savings may be made in their implementation. While no formal economic evaluations of school-based interventions for DRV and GBV have yet been conducted, the evidence suggests that interventions that reduce the incidence of violent events may save more money than they require to implement.

## Results

### Included Studies

Following de-duplication, the full review search identified 40,160 records that were screened on title and abstract, and 788 records were screened on full text. Seven studies (Bush et al., 2018; Cissner & Ayoub, 2014; Crooks et al., 2017; Jones et al., 2021; Luo et al., 2020; Meiksin et al., 2020; Wolfe et al., 2009) reporting cost and/or resource use data for eight interventions were identified. The review did not identify any studies that included a formal evaluation of the cost-effectiveness of interventions. A PRISMA diagram for the review is provided in the supplementary material.

Five (Bush et al., 2018; Cissner & Ayoub, 2014; Jones et al., 2021; Meiksin et al., 2020; Wolfe et al., 2009) of the included studies were based on RCTs, and two studies (Crooks et al., 2017; Luo et al., 2020) were based on observational case studies of the intervention. All seven studies involved students between 11 and 17 years of age and all were conducted in the school setting. Four studies (Bush et al., 2018; Cissner & Ayoub, 2014; Jones et al., 2021; Luo et al., 2020) were conducted in the United States, two studies (Crooks et al., 2017; Wolfe et al., 2009) were conducted in Canada, and one study (Meiksin et al., 2020) was conducted in the United Kingdom. One study reported results for two interventions (Luo et al., 2020).

An overview of the included studies is provided in Table 1. Because studies were primarily descriptive and were not “full” economic evaluations, we did not undertake formal appraisal, but we did note variations in methods across studies and consider where these may have affected outcomes.

**Table 1.** Overview of Included Studies.

Lead author and date	Study design	Study methods	Location	Demographic details	Age groups (% male)	Sample size
Bush et al. (2018)	Cluster RCT	Costs classified as start-up or ongoing. Interviews and analysis of budgets used to collect data. Educators and supervisors asked to estimate time spent on activities related to the program.	USA, Kentucky	26 schools. 52% eligible for reduced/free school meals. >80% White. Average graduation and college enrolment rates were 73.9% and 57.5%, respectively.	14–18 (NR)	28 educators, 2559 students
Cissner & Ayoub (2014)	Cluster RCT	‘Start Strong Bronx’ provided cost estimates for the experimental schools in the study. Methods used unclear.	USA, The Bronx (NYC)	Grade 7 students from 13 urban public middle schools. Predominantly Hispanic (73%) and Black (30%) from low income households. Economic need index between 0.76–1.06. 80% heterosexual, 5% bisexual and 15% uninterested in dating/sex. 8% sexually active, 57% had ever dated, 45% dated in the last 3 months.	12 (45%)	709 students
Crooks et al. (2017)	Case studies	Presented 4 case studies in different geographical regions and in different stages of program implementation. Quantified both costs and benefits of the intervention in US dollars.	Canada, Southern Ontario, Canadian Northwest Territories, Alberta	Grade 7, 8, and 9 students. Demographic characteristics varied across the four case studies.	12–17 (NR)	20803
Jones et al. (2021)	Secondary data analysis of two RCTs	Authors used rates of dating abuse to estimate the number of events that had been avoided as a result of the intervention. Costs avoided are based solely on sexual assaults avoided rather than other kinds of dating abuse.	USA, California and Pennsylvania	Urban public high schools in California, mix of public and private middle schools in Western Pennsylvania. Only included male participants in organized sports.	11–18 (100%)	2493
Luo et al. (2020)	Observational study	Estimated implementation costs across 4 sites over 4 years based on data from local public health departments and contractors funded by CDC. Also used known/estimated material costs from 2019. Salary estimates taken from each site/year during demonstration project. Cost estimates based on public health departments and contractors	USA, Alameda County, Baltimore, Broward County and Chicago	6^th^-, 7^th^-, and 8^th^-grade students across four large urban areas. Most were Black (55%) or Hispanic (28%).	11–14 (NR)	Range of student numbers by year and site: Dating Matters – 599–6641 Safe Dates – 146–1968
Meiksin et al. (2020)	Pilot RCT	Costs for NSPCC trainer and school staff time taken from employer. Unit costs of health services taken from NHS Reference Costs, British National Formulary, New Economy Manchester Unit Cost Database and Unit Costs of Health and Social Care. Unit costs for criminal justice taken from U.K. Home Office. Accounted for resource including trainer travel, classrooms required, and costs to cover teachers during training.	England	Year 9 and 10 students from four state schools in southern England with varying levels of deprivation (mean income deprivation affecting children index score of 0.23). The majority (46.8%) of students were white British, 39.9% reported no religion while 22.8% were Christian, 71.7% had some dating experience and 44% had a partner in the last 12 months, 84.2% were heterosexual, 2.7% homosexual and 5.1% bisexual.	14–17 (51.5%)	1529 students
Wolfe et al. (2009)	Cluster RCT	Used cost of teacher release time for 1 day of training plus the cost of curriculum and video resources. Also included costs of incentives.	Canada, Southwest Ontario	Grade 9 students in 20 public schools with a mix of rural and urban areas. Predominantly White and from two-parent households. 60% had dated in the previous 12 months.	14–15 (47%)	1722

*Note.* CDC = Centers for Disease Control and Prevention; NHS, National Health Service; NR = not reported; NSPCC = National Society for the Prevention of Cruelty to Children; NYC = New York City; RCT = randomized controlled trial.

### Interventions Evaluated

A brief overview of the interventions evaluated is provided in the supplementary material. Four interventions targeted DRV (Crooks et al., 2017; Luo et al., 2020; Wolfe et al., 2009), and four interventions (Bush et al., 2018; Cissner & Ayoub, 2014; Jones et al., 2021; Meiksin et al., 2020) targeted both DRV and GBV. One intervention was evaluated in more than one study (The Fourth R, *n* = 2; Crooks et al., 2017; Wolfe et al., 2009). Five of the interventions were facilitated by teachers, one (Jones et al., 2021) by athletic coaches, and one (Bush et al., 2018) by staff from a rape crisis center. The majority of the interventions involved teaching students within classroom settings (Cissner & Ayoub, 2014; Crooks et al., 2017; Luo et al., 2020; Meiksin et al., 2020; Wolfe et al., 2009) or teaching delivered within alternative settings (e.g., athletic coaching sessions [Jones et al., 2021]) and methods (e.g., peer delivery [Bush et al., 2018]). Interventions all required some degree of training for those delivering the intervention. Several interventions included additional components, including facilitating role-play (Bush et al., 2018; Crooks et al., 2017; Wolfe et al., 2009), digital materials (Crooks et al., 2017; Luo et al., 2020; Meiksin et al., 2020; Wolfe et al., 2009), and handouts (Crooks et al., 2017; Wolfe et al., 2009). Three of the interventions also included information or training for parents of the student participants. The follow-up duration of the studies varied from 15 months to 4 years.

### Results of Included Studies

The results are presented in Table 2. Six (Bush et al., 2018; Cissner & Ayoub, 2014; Crooks et al., 2017; Luo et al., 2020; Meiksin et al., 2020; Wolfe et al., 2009) studies reported the costs and resource use associated with their respective interventions, and two studies (Crooks et al., 2017; Jones et al., 2021) reported cost savings from the estimated reduction in DRV predicted by the efficacy of the intervention within the evaluation.

**Table 2.** Costs and Resource Use Reported by Included Studies.

Lead author and date	Intervention	Itemized costs		Total costs	Materials	Training
Bush et al. (2018)	Green-dot bystander intervention	Cost for 13 schools: Cost of purchasing program and training two people to train educators - $20,000 (first 2 years only) Consulting fee - $4,500 per year (first 4 years only) Travel - $15,360 per year Supplies - $11,300 per year Coaching of subset of children - $25,000 per year (no coaching in year 1) Educators—range from $254,470–$284,407 per year Time—educators/supervisors spent a median of 37.5/45.0 hours, respectively, over the school year		$1.6 million over 4 years. $123,735 per school ($49.93 per student) over 5 years	$11,300 per year. Presumably not reusable as the same cost is incurred each year	$20,000 (for first two years only, no ongoing cost)
Cissner & Ayoub (2014)	Fourth R Curriculum	Costs according to creators: Teacher Binder (includes cards, a DVD with role-play examples, two DVDs with skills for effective relationships, and a CD-ROM with handouts, overheads, a unit test, and other resources for printing) - $135 each 4 DVDs - $325 per set Youth Safe Schools Committee Manual - $25 each 1-day, off-site teacher training workshop - $150 per person 1-day, on-site teacher training workshop - $1,500 per 25 participants (plus trainer travel expenses) 1.5-day, on-site master trainer training - $12,500 for 25 participants plus trainer travel expenses Master Trainer Manual (includes fidelity checklists) - $150 each 2-day, on-site consultation - $2,000 (plus travel expenses) Phone and email support—free Student Satisfaction Questionnaire—free Teacher Implementation Questionnaire—free	Actual cost for 10 schools: 20 binders: $75/each × 20 = $1,500 15 sets of materials: $90 per set × 15 = $1,350 20 teachers paid to attend training: $95 × 20 teachers = $1,900 Food for teacher training: $1,000 Master Trainers: $1,015 Time—teacher training was 6 hours. Delivery was 26 hours. Unclear whether further time was taken for preparation or other activities.	$12.21 per student or $676 per school per year	$2,850 (per 20 teachers)	$3915 per 20 teachers. 6-hour duration.
Crooks et al. (2017)	Fourth R Curriculum	Costs over first 5 years (115 students in years 1 and 2, 265 in year 3, and 416 in years 4 and 5): Adaptation of curriculum to include local perspective—CA$5,000 Development of localized video resources (3 videos/locations)—CA$21,350 Materials—hard copies—CA$19,100 Materials—e-licensing copies—CA$1,600 Trips for national education coordinator—CA$40,000 Other trips—CA$20,000 Coordinator—CA$50,000 Focus groups and report writing—CA$10,000 Developing master trainers—CA$4,630		CA$129/student in first 5 years in most expensive of the three case studies. Reducing to CA$2-$33 per student going forward (varies by case study).	CA$20,700 over first 5 years (for Northwest Territories subgroup)	Varied across 5 years and 3 locations from CA$14,625 to CA$151,575
Jones et al. (2021)	Coaching boys into men	NR		NR	NR	Training of coaches = 0.5 days Ongoing support and technical assistance = approximately one hour per week per school.
Luo et al. (2020)	Dating matters	Prevention lead 1.0 FTE^a^ - $55,000–$85,000 DM coach 0.5 FTE^a^ - $17,500–37,500 Policy lead 0.1 FTE^a^ - $5,500–$8,500 Data collection lead 0.1 FTE^a^ - $5,500–$8,500 Staff at lead organization - $93,500–$134,500 Youth program facilitator - $0–$55,000 Parent program facilitator 0.5 FTE^a^ - $7,500–$27,500 I2i youth communication program - $8,750–$13,750 Printed materials, 6th grade - $195–$2,249 7th grade - $173–2,364 Coach materials - $3 Facilitator materials - $3 Parent materials - $21–$395		$175,452 per school ($145.40 per student).		Not included as it is now available free online. Took “10 hours or less.”
	Safe dates	Youth program facilitator 0.33 FTE^a^ - $0–$18,333 Curricula - $245 (first year only) Facilitator guide - $7 Student handouts - $219–$2,952		$12,148 per school per year ($38.14 per student).		
Meiksin et al. (2020)	Safe dates	NSPCC-delivered training travel and delivery time – 19 hours 13 minutes, £31.07 hourly cost of trainer time Teach time for training (on average 8 teachers per school) – 3 hours 38 minutes, £31.15 hourly cost of teacher time All staff training (on average 76 staff) – 47 minutes, average of £21.86 hourly rate Trainer preparation and delivery – 7 hours 17 minutes, £31.15 hourly cost		NR		£3030.80 per school (thought to be underestimate). Mean duration of 47 minutes
Wolfe et al. (2009)	Fourth R Curriculum	Teacher training – 6 hours (CA$200 per teacher) Intervention delivery – 28 hours Materials—mean of CA$700 per school or CA$175 per teacher		CA$16 per student in first year (one-time costs)	Mean of CA$700 per school or CA$175 per teacher (reusable)	CA$200 per teacher to free-up teachers for training. Single 6 hour workshop

*Note.* DVD = digital versatile disk; CD-ROM = compact disk read-only memory; DM, Dating Matters; FTE, full-time equivalent; NR = not reported; NSPCC = National Society for the Prevention of Cruelty to Children.

a Assumes a 40-hour working week.

#### Cost and Resource Use of Included Interventions

The total cost of interventions, where reported, ranged from US$12 to $145 per student. Staff costs for delivering the intervention (either training school staff or else hiring external teams to deliver) were generally the highest cost, and variation in the involvement and training of staff was a major driver of differences in costs between studies. Several studies noted one-off material costs that would be incurred in the first year of implementation, which would result in lower costs in subsequent years and once the costs of staff training were accounted for. However, re-training staff or training new staff in schools with a high staff turnover continued to be a significant driver of costs. It was generally more expensive to hire external staff than train internal staff.

Materials, such as handouts, DVDs, and course manuals, were costly. Not all studies reported whether materials were reusable in subsequent years of the intervention, although Crooks et al. (2017) reported that freely accessible materials that schools were able to print themselves were associated with a reduction in costs. Adapting materials to suit local contexts was particularly expensive, one study (Crooks et al., 2017) reported this to cost CA$26,350. One of the interventions evaluated, Project Respect (Meiksin et al., 2020), involved a change at the school level, including a review of school policies and increased monitoring of students by school staff, and did not show an accompanying increase in costs compared with other interventions.

Two studies (Bush et al., 2018; Crooks et al., 2017) demonstrated that intervention costs per student can be reduced where they can be shared across a larger number of students. Crooks et al. (2017) found that costs were increased as a result of smaller class sizes and the distribution of schools over a larger geographical area. Similarly, Bush et al. (2018) reported that while costs to implement Green Dot over the first 5 years were $1.6 million for 13 schools (i.e., $123,000 per school), the cost of subsequently adding another school was $25,510 as there would be no start-up costs.

#### Cost Savings of Interventions

Crooks et al. (2017) estimated savings of CA$32 per student for reduced physical dating violence, and CA$1,978 per student in avoided costs of violent delinquency in the long term after receiving the Fourth R intervention. Jones et al. (2021) estimated that the implementation of Coaching Boys into Men avoided 85 dating abuse incidents in high school students and 49 in middle school students over the course of 3 months. Across 1000 students, this was considered to result in estimated savings of $2.5 million, or $2,500 per student.

## Discussion

High-quality economic data for school-based interventions are crucial for guiding educational policy, particularly in state-funded schools where budgets are tightly controlled and policymakers are required to justify the opportunity costs of spending. However, despite the comprehensive literature search strategy used, this systematic review did not identify any cost-effectiveness evaluations of school-based interventions for DRV or GBV. As part of the broader review, 68 RCTs and 108 observational studies evaluating DRV or GBV interventions were identified, but only a handful of these studies reported any cost or resource-use data.

As with evaluations of other school-based interventions (e.g., for physical activity policy [Lane et al., 2022]), the largest cost of interventions related to staff costs, including training and/or hiring of external organizations to deliver interventions. Costs were shown to increase in settings where staff turnover is high due to the need to re-train staff, and costs were shown to reduce where these could be shared among a larger group of students. This means that in comparison, interventions targeting “high-risk” students could be more costly to implement, and to date, there is relatively little evidence on the effectiveness of such an approach.

Provided that the costs of diverting school staff away from other responsibilities are manageable, it is likely that interventions delivered “in-house” will be less costly, as the costs of training staff will be shared over multiple years of implementation. There may also be broader benefits of school staff delivering the intervention, for example, their greater familiarity with students may help with intervention delivery (Luo et al., 2020), and staff knowledge may translate to other areas of their role in schools.

Increasing the scale of interventions, to broader age groups or across multiple schools, may also lead to cost savings through shared staff training and the reuse of materials. The diffuse benefits of interventions for DRV and GBV, for example, through ongoing transmission of knowledge, may also be greater with wider implementation (Cissner & Ayoub, 2014). However, it is common for the developers of interventions to encourage schools to adopt interventions according to the age, baseline risk, and cultural background of students. This may limit the ease with which resources are shared by schools, and one study in the review showed that the costs of adapting interventions to cultural contexts may significantly increase costs (Crooks et al., 2017).

The costs of implementing school-based interventions for DRV may ultimately be justified if these are able to reduce the number of DRV incidents. While estimates of cost savings may be somewhat unreliable when the longer-term effectiveness for reducing DRV and GBV incidents is lacking, there are substantial cost and health benefits to reducing these incidents. Such events are highly costly due to the costs they present to law enforcement agencies and the costs incurred to society through their impact on people’s health and employment (Walby, 2009). When delivered within the school context, interventions for DRV and GBV may result in higher cost savings over students’ lifetimes than those implemented in adults later in life. Cost savings of interventions will largely benefit budgets for sectors outside of education, and therefore policymakers may wish to consider the onward benefits of spending on DRV and GBV prevention within schools.

The findings of this review are limited by the paucity of studies that have evaluated costs associated with school-based interventions for DRV and GBV, and by the variability in units and categorization of reported costs, which limits comparison. It is also possible that the findings may have limited generalizability beyond the settings in which the included studies took place, for example, to low- and middle-income countries, where no evidence was identified.

Nevertheless, the studies presented herein provide various key learnings for the costs of interventions for DRV and GBV. Decision makers should consider the potential staffing costs for implementing interventions, and how collaborations with other schools and districts may increase feasibility. Education policy toward reducing violence in schools may also consider the potential value of long-term reductions in costs associated with violence in other public sectors. Economic evaluations of interventions found to be effective in reducing violence behavior are needed and would make a stronger case for schools to adopt interventions for DRV and GBV.

## Supplemental Material

sj-docx-1-heb-10.1177_10901981221138064 – Supplemental material for Systematic Review of the Economics of School-Based Interventions for Dating Violence and Gender-Based Violence Click here for additional data file. Supplemental material, sj-docx-1-heb-10.1177_10901981221138064 for Systematic Review of the Economics of School-Based Interventions for Dating Violence and Gender-Based Violence by Fraizer Kiff, Naomi Shaw, Noreen Orr, Andrew. J. Rizzo, Annah Chollet, Honor Young, Emma Rigby, Ann Hagell, Vashti Berry, Chris Bonell, G. J. Melendez-Torres and Caroline Farmer in Health Education & Behavior
