# Supplementary material for: Systematic Review of the Economics of School-Based Interventions for Dating Violence and Gender-Based Violence
Source: Health Educ Behav. 2022 Dec 12;50(3):339–46. doi: 10.1177/10901981221138064 (PMC10164611; doi:10.1177/10901981221138064)
Supplement: sj-docx-1-heb-10.1177_10901981221138064 – Supplemental material for Systematic Review of the Economics of School-Based Interventions for Dating Violence and Gender-Based Violence [file sj-docx-1-heb-10.1177_10901981221138064.docx]

# Supplementary material

## Literature search strategy

### Bibliographic database searches

The following bibliographic databases were searched in January 2020 and updated in June 2021. An example database search strategy (for Ovid MEDLINE) is also provided below. The search strategies were developed by an experienced Information Specialist following extensive scoping searches and consideration of existing systematic reviews. The search terms used included a combination of free-text terms and subject headings for topic areas of interest. No study design filters were applied, and searches were not restricted by date or language of publication. The bibliographic database searches were revised to increase precision for the update search in June 2021. The revised strategy was based on analysis of titles, abstracts and index terms of included studies. The updated search strategy also incorporated programme names not identified in the initial scoping searches. Results from the update searches were not date limited, but instead de-duplicated against previous result sets to ensure records were not missed.

- MEDLINE, Embase, PsycINFO, Social Policy and Practice (Ovid);
- CINAHL, ERIC, British Education Index, Education Research Complete, EconLit, Criminal Justice Abstracts (EBSCO);
- Cochrane Database of Systematic Reviews (CDSR) and the Cochrane Central Register of Controlled Trials (CENTRAL);
- NHS Economic Evaluation Database (NHS EED via the Centre for Reviews and Dissemination);
- Social Science Citation Index and Conference Proceedings Citation Index (Web of Science, Clarivate Analytics);
- Australian Education Index, ProQuest Dissertations & Theses Global, Sociological Abstracts including Social Services Abstracts, Applied Social Sciences Index and Abstracts (ProQuest);
- Trials Register of Promoting Health Interventions (TRoPHI) and Bibliomap (EPPI-Centre);
- Campbell Systematic Reviews (Campbell Collaboration).

Ovid MEDLINE search strategy (June 2020)

1 exp Intimate Partner Violence/ (9469)

2 Gender-Based Violence/ (188)

3 Stalking/ (203)

4 Rape/ (6264)

5 Sex Offenses/ (8867)

6 Battered Women/ (2620)

7 Spouse abuse/ (7352)

8 Coercion/ (4594)

9 Domestic violence/ (6375)

10 Homophobia/ (518)

11 (stalking or stalker*).ti,ab. (792)

12 rape*.ti,ab. (11749)

13 "intimate partner violence".ti,ab. (7375)

14 IPV.ti,ab. (6263)

15 (gender* adj3 violen*).ti,ab. (1623)

16 GBV.ti,ab. (1120)

17 SRGBV.ti,ab. (1)

18 (domestic adj3 (abuse* or abusive or aggressi* or assault* or attack* or bully* or coerc* or cyberbully* or femicid* or harass* or homicid* or injur* or manipulate* or murder* or rape* or threaten* or violen* or victimi?ation or revictimi?ation or re-victimi?ation)).ti,ab. (6880)

19 "violence against women".ti,ab. (2369)

20 ((date or dating) adj3 (abuse* or abusive or aggressi* or assault* or attack* or bully* or coerc* or cyberbully* or femicid* or harass* or homicid* or injur* or manipulate* or murder* or rape* or threaten* or violen* or victimi?ation or revictimi?ation or re-victimi?ation)).ti,ab. (2532)

21 ((relationship* or partner* or acquaintance* or non-stranger* or nonstranger*) adj3 (abuse* or abusive or aggressi* or assault* or attack* or bully* or coerc* or cyberbully* or femicid* or harass* or homicid* or injur* or manipulate* or murder* or rape* or threaten* or violen* or victimi?ation or revictimi?ation or re-victimi?ation)).ti,ab. (17167)

22 ((boyfriend* or boy-friend* or girlfriend* or girl-friend*) adj3 (abuse* or abusive or aggressi* or assault* or attack* or bully* or coerc* or cyberbully* or femicid* or harass* or homicid* or injur* or manipulate* or murder* or rape* or threaten* or violen* or victimi?ation or revictimi?ation or re-victimi?ation)).ti,ab. (47)

23 (interpersonal adj3 (abuse* or abusive or aggressi* or assault* or attack* or bully* or coerc* or cyberbully* or femicid* or harass* or homicid* or injur* or manipulate* or murder* or rape* or threaten* or violen* or victimi?ation or revictimi?ation or re-victimi?ation)).ti,ab. (2416)

24 (sexual* adj3 (abusive or aggressi* or assault* or attack* or bully* or coerc* or cyberbully* or femicid* or harass* or homicid* or injur* or manipulate* or murder* or rape* or threaten* or violen* or victimi?ation or revictimi?ation or re-victimi?ation)).ti,ab. (15390)

25 ((coerc* or forced or unwanted or nonconsensual or non-consensual) adj2 sex*).ti,ab. (2147)

26 (grope or groped or groping).ti,ab. (144)

27 (sext or sexts or sexting).ti,ab. (206)

28 (homophobi* or transphobi* or biphobi* or homonegativ*).ti,ab. (1662)

29 ((LGB or LGBT* or homosexual* or lesbian* or gay or bisexual* or queer* or transgender* or transsexual*) adj3 (abuse* or abusive or aggressi* or assault* or attack* or bully* or coerc* or cyberbully* or femicid* or harass* or homicid* or injur* or manipulate* or murder* or rape* or threaten* or violen* or victimi?ation or revictimi?ation or re-victimi?ation)).ti,ab. (697)

30 "long live love".ti,ab. (4)

31 (greendot or "green dot").ti,ab. (28)

32 "project respect".ti,ab. (27)

33 ("Media Aware" or mediaaware).ti,ab. (5)

34 TakeCARE.ti,ab. (14)

35 "Fourth R".ti,ab. (24)

36 "Safe Dates".ti,ab. (22)

37 "Shifting boundaries".ti,ab. (49)

38 "Teen choices".ti,ab. (4)

39 "good schools toolkit".ti,ab. (2)

40 "mentors in violence prevention".ti,ab. (5)

41 "Expect Respect".ti,ab. (8)

42 "Second Step".ti,ab. (10890)

43 SS-SSTP.ti,ab. (1)

44 "It's your game".ti,ab. (12)

45 DaVIPoP.ti,ab. (0)

46 (Benzies adj2 Batchies).ti,ab. (1)

47 or/1-46 (80507)

48 Schools/ (37880)

49 exp School Health Services/ (22957)

50 Students/ (58229)

51 Curriculum/ (74944)

52 school*.ti,ab,jw. (290890)

53 (pupil or pupils).ti,ab. (21829)

54 (classroom* or class-room*).ti,ab. (17106)

55 or/48-54 (420474)

56 47 and 55 (5600)

### Additional searches

We searched trial registers to identify ongoing or unpublished research (clinicaltrials.gov, WHO ICTRP) and we conducted searches for grey literature including conference abstracts, reports and theses from web searches, repositories of grey literature (e.g. OpenGrey.eu) as well as searches of websites identified in initial scoping searches (including VAWnet; [www.vawnet.org](http://www.vawnet.org)).

In addition, the reference lists of existing systematic reviews or relevant reports were reviewed for relevant literature. Forward and backward citation chasing was conducted on included studies identified from the June 2020 bibliographic database searches. Scopus (Elsevier), Web of Science (Clarivate) and Google Scholar were used for citation chasing, and bibliographies of included studies were manually checked where this information was incomplete on Web of Science and Scopus.

Targeted searches were conducted in Web of Science and Scopus using first and last author names for studies identified in bibliographic database searches in June 2020. Specific project names (for e.g. Project Respect, Shifting Boundaries or Safe Dates) were included in the update search strategies in bibliographic databases, and Google Scholar. Results were screened in Google Scholar, with the first 200 records scanned for each search string. Websites identified in initial scoping searches were browsed or searched for additional reports (including VAWnet: [www.vawnet.org](http://www.vawnet.org); USAID: [www.usaid.gov](http://www.usaid.gov); AVA – Against Violence and Abuse; UNGEI, National Criminal Justice Reference Service: [www.ncjrs.gov](http://www.ncjrs.gov)).

## PRISMA diagram

Records identified from database searching (n=54,064)

Duplicate records removed before screening

(n=28,048)

Records screened at title/abstract

(n=26,016)

Records excluded

(n=25,517)

Reports sought for retrieval

(n=499)

Reports not retrieved

(n=1)

Reports assessed for eligibility (n=498)

searching)

Reports excluded (n=369)

Studies included in review:

outcome evaluations (n=68); process evaluations (n=137);

economic evaluations (n=0);

costing and resource use (n=7);

mediation/moderation analyses (n=61)

from total reports (n=247)

Records identified from:

- Website searches (n=10)
- Google Scholar searches (n=26)
- Citation chasing (n=18,571)
- Reference lists of existing systematic reviews (n=134)
- First-last author searching (n=18,941)

Records screened at title/abstract

(n= 14,144)

Records excluded (n=13,838)

c

Reports sought for retrieval

(n=306)

Reports not retrieved

(n=16)

Reports assessed for eligibility

(n=290)

Reports excluded (n=172)

## Overview of included interventions

Table 3: Description of interventions

| **Lead author and year** | **Intervention** | **Intervention target (DRV or GBV)** | **Duration of intervention** | **Intervention Summary** |
| --- | --- | --- | --- | --- |
| Bush 2018 (Bush et al., 2018) | Green-Dot | Both | 5 years. One student assembly/speech each year, followed by 4-6 hours’ of training with selected students. | Aimed to engage potential bystanders to act to reduce sexual violence and related forms of interpersonal violence. Male and female students were trained to recognise situations and behaviours that can contribute to violence and determine actions they could safely take to reduce the likelihood or effect of violence. The majority of target students in the school (>50%) received an introductory 'persuasive' speech from an external speaker on the role of bystander intervention and ‘green’ vs. ‘red dot’ behaviours. Then teachers select 10-15% of students they considered to be 'leaders' in the school who received training (5 hours) to recognise situations and behaviours that can contribute to violence and determine actions they could safely take to reduce the likelihood or effect of violence. |
| Cissner 2014 (Cissner & Ayoub, 2014) | Stay Strong Bronx (adaptation of the Fourth R) | Both | 21x 45-minutelessons | Stay Strong Bronx is an adaptation of The Fourth R, which is a program designed to promote healthy behaviours related to dating, sexual behaviour, bullying, and substance use. In this version, the program was adapted for shorter 45-minute lessons, and was delivered in both sex-segregated and sex-mixed classes. The wording in classes was also adapted for 7th grade students and to make it more relevant to an 'urban' population. |
| Crooks 2017 (Crooks et al., 2017) | The Fourth R | DRV | 21 – 22 x 75-minute lessons | The Fourth R is a program designed to promote healthy behaviours related to dating, sexual behaviour, bullying, and substance use. This version was conducted with grade 7, 8 and 9 students, and comprised three units to address violence, substance use, and healthy sexuality/sexual behaviour. Limited information was reported about the intervention as delivered in this evaluation, and it was assumed that the intervention was consisted with the original manual. According to this, sex-segregated classes were delivered by teachers, guided by detailed lesson plans, video resources, role-play exercises, rubrics, and handouts were provided for all lessons. Teachers received a 6-hour training workshop taught by an educator and a psychologist to review the materials and participate in skill-building exercises for engaging youths. Information was provided for parents during grades 7, 8 and 9 orientation and in the form of 4 newsletters. Student-led “safe school committees” were established. |
| Wolfe 2009 (Wolfe et al., 2009) | The Fourth R | DRV | 21 x 75-minute lessons | The Fourth R is a program designed to promote healthy behaviours related to dating, sexual behaviour, bullying, and substance use. This version was conducted with grade 9 students, and comprised three units to address violence, substance use, and healthy sexuality/sexual behaviour. Limited details were provided about the intervention delivered in this evaluation, and therefore it was assumed that the intervention was delivered according to the manual. Accordingly, sex-segregated classes were delivered by teachers, guided by detailed lesson plans, video resources, role-play exercises, rubrics, and handouts were provided for all lessons. Teachers received a 6-hour training workshop taught by an educator and a psychologist to review the materials and participate in skill-building exercises for engaging youths. Information was provided for parents during grade 9 orientation and in the form of 4 newsletters. Student-led “safe school committees” were also established. |
| Jones 2021 (Jones et al., 2021) | Coaching boys into men | Both | 12 topics covered over 3 months | Coaching Boys into Men (CBIM) is a dating abuse and sexual violence prevention program delivered by athletic coaches to male student athletes. Coaches received a 60-minute training session, and eleven ‘training cards’ to guide brief (10-15-minute) weekly discussions with athletes. Topics included respectful language and behaviour, encouraging intervention when witnessing peers’ harmful behaviour, preventing DRV, and challenging ‘hyper-masculine’ behaviours. An advocate was available to assist coaches with concerns that arise during program delivery, including disclosures. |
| Luo 2020 (Luo et al., 2020) | Dating Matters | DRV | 3 years with 7 session in 6^th^ and 7^th^ grade and 10 sessions in 8^th^ grade. | Dating Matters is a multi-component DRV prevention intervention. In classrooms 6^th^ – 8^th^ grade students received classes about healthy relationships and assisted youth in practicing healthy relationship skills. Training is also provided for parents and teachers/school personnel. A youth communications program was established that reinforced messaging about healthy relationships using ‘near-peer’ ambassadors with community activities, printed materials, and digital resources. Local health departments were also assisted to assess and build capacity for DRV prevention, including tracking local policy and indicator data. The intervention comprised delivery by both external and internal staff, with peer delivery. |
| Luo 2020 (Luo et al., 2020) | Safe Dates | DRV | 10x 45-minute classes in 8^th^ Grade. | Safe dates is an intervention addressing attitudes and behaviours associated with DRV. Typically includes a theatre program and training for parents, though limited information described for this evaluation. Program materials were sent to parents, but these were not described. |
| Meiksin 2020 (Meiksin et al., 2020) | Project Respect | Both | 2 years, including 8 class-based lessons. | A manualised, multicomponent, DRV intervention implemented by the NSPCC. Training was provided by the NSPCC for governors and key school staff to enable them to plan and deliver the intervention in their schools, review school policies to help reduce gender-based harassment and DRV, and increase staff presence in ‘hotspots’ for these behaviours. Staff who received the training subsequently trained other school staff in safeguarding to prevent, recognise and respond to gender-based harassment and DRV. Written information was provided for parents on the intervention and advice on preventing and responding to DRV. The Circle of 6 app, with the appearance of a games app, was launched to help individuals contact support if threatened by or experiencing DRV. A classroom curriculum delivered by teachers to students aged 13–15 years, including student-led campaigns. |

Abbreviations: DRV, dating related violence; GBV, gender-based violence
